# Strategies for recruitment of adolescent girls into physical activity programmes: a systematic review and regression analysis

**DOI:** 10.1186/s12889-026-27261-z

**Published:** 2026-04-11

**Authors:** Tanya O’Brien, Catherine D Darker, David Mockler, Emer M Barrett

**Affiliations:** 1 Discipline of Physiotherapy, School of Medicine, The University of Dublin, Trinity College Dublin, St James’s Hospital, James Street, Dublin 8, Dublin, D08 NHY1 Ireland; 2 Discipline of Public Health and Primary Care, Institute of Population Health, School of Medicine, The University of Dublin, Trinity College Dublin, Russell Centre, Tallaght Cross, Dublin, D24 DH74 Ireland; 3 John Stearne Medical Library, School of Medicine, The University of Dublin, Trinity College Dublin, St James’s Hospital, James Street, Dublin 8, Dublin, D08 NHY1 Ireland

**Keywords:** Physical activity, Adolescent girls, Recruitment, Recruitment strategies, Systematic review, Randomised controlled trials

## Abstract

**Background:**

Globally, adolescent girls are one of the least active populations, with ~ 15% meeting WHO physical activity (PA) guidelines. This review offers the first quantitative synthesis of recruitment rates into PA randomised controlled trials targeting adolescent girls and identifies strategies which appear most promising, providing actionable guidance for PA providers and researchers.

**Methods:**

Five databases were searched, supplemented by citation hand-searches. Eligible studies were randomised controlled trials of girls aged 10–19 years with PA interventions lasting ≥ 4 weeks that reported recruitment rates or pre-determined recruitment goals; studies limited to clinical populations or athletes were excluded. Descriptive statistics were used to calculate mean recruitment rates overall and by subgroups such as age, socioeconomic status, and programme setting, while correlation and regression analyses examined associations between recruitment strategies and recruitment outcomes.

**Results:**

Fifteen thousand one hundred records were identified; 37 through citation search. After removing duplicates (*n* = 8,603), 6,407 records were screened by title and abstract and 134 full texts assessed. Following consensus, 27 studies were included; 23/27 were school-based. The mean recruitment rate was 62.6% (± 27.8), decreasing to 56.5% (± 35.0) in low-SES populations. Incomplete reporting of the recruitment funnel was common. Recruitment was higher amongst younger (65.9% ± 26.7) versus older (51.2% ± 32.1) girls. Teacher involvement was associated with improved recruitment: when teachers were financially supported to organise activities (R^2^ = 0.44, *p* = 0.005) or followed up for consent with absent girls (R^2^ = 0.25, *p* = 0.048). Recruitment goals were achieved in 68% of studies, particularly those using researcher presentations or PA taster sessions (*r* = 0.52, *p* = 0.041). School-based programmes during class time recruited more effectively (62.3% ± 28.1) than those offered at optional times (54.9% ± 30.9). Incentives for girls was associated with the lowest recruitment rates (51.2% ± 32.1) and reduced retention (R^2^ = 0.39, *p* = 0.024). Mean retention was high (84.9% ± 13.3); better in school-based (86.0% ± 12.6) versus non-school-based (81.2% ± 16.3) programmes and improved when fitness assessments were included (R^2^ = 0.24, *p* = 0.020).

**Conclusion:**

Recruiting adolescent girls into PA programmes is feasible but challenging. Teacher engagement, integrating PA into school timetables, and interactive approaches (presentations, tasters) emerged as potentially promising strategies for recruiting girls, though the evidence base was predominantly school-based and reporting of recruitment strategies was often incomplete.

**Supplementary Information:**

The online version contains supplementary material available at 10.1186/s12889-026-27261-z.

## Introduction

Physical activity (PA) is vital for youth health, and yet only 15% of adolescent girls worldwide meet the World Health Organization (WHO) guideline of at least an average of 60 min of moderate-to-vigorous physical activity per day [1]. A recent analysis across 146 countries confirmed a 7% gender gap in PA participation, with girls consistently less active than boys [2]. Regular exercise supports physical and mental health, boosting self-esteem and academic performance while reducing the risk of chronic diseases [3, 4], and since PA habits established in adolescence often persist into adulthood, these inactive girls may face increased long-term health risks [5]. Furthermore, already-poor adolescent PA levels may have been even more negatively impacted by the COVID-19 pandemic [6, 7], emphasising the urgency of effective and scalable interventions.

Rising awareness of this inactivity crisis in adolescent girls has led to the development of innovative PA programmes, many of which are co-designed with girls and grounded in behaviour change theory [8–11]. Despite these efforts, recruiting adolescent girls into PA programmes remains a major challenge [8, 12–14], and girls cannot reap the benefits of even the best-designed PA programmes if they do not join in the first place. Programme providers report enrolment of just 10–29% of eligible girls even with multiple recruitment efforts [8, 10], often requiring adaptation of recruitment strategies [11, 15], or failing to meet their pre-determined recruitment goals [12, 16]. These challenges highlight recruitment as a critical, yet underexamined, determinant of programme reach and impact.

Qualitative studies have previously explored adolescent girls’ perceptions of PA and barriers/facilitators to participation [17–20], however, quantitative evidence on expected recruitment rates and which recruitment strategies are associated with improved recruitment remains limited. Moreover, while we know that higher socioeconomic status (SES) in adolescents is associated with higher PA levels [21], the influence of SES on recruitment into PA in adolescent girls has not been explored. This systematic review aimed to investigate the recruitment of adolescent girls into randomised controlled trials (RCTs) of PA programmes, with a secondary analysis of retention.

The objectives of this review were threefold: first, to establish recruitment rates for adolescent girls in PA intervention RCTs and examine how these varied by subgroups such as SES and age; second, to synthesise the strategies programme providers and researchers have actually used to recruit this cohort; and third, to examine associations between recruitment strategies and recruitment outcomes in practice. This review considers not only how many girls sign up, but also which approaches appear most promising in supporting recruitment.

## Methods

A quantitative systematic review was completed following the Preferred Reporting Items for Systematic Reviews and Meta-Analysis (PRISMA) guidelines [22, 23] and reported using the PRISMA 2020 checklist [24] (see Additional files Item 1). The review was registered with PROSPERO (registration number CRD#42023475858) and the protocol was previously published [25].

### Eligibility criteria

The eligibility criteria for inclusion in this review were based on the PICOTS Framework (Population, Intervention, Comparison, Outcome, Time frame, Study Type) [26]. 

### Population

Studies that involved adolescent girls aged 10–19 (per WHO’s definition of adolescents [27]) were included. Studies limited to special health populations (e.g., Type 1 diabetes) or athletes were excluded, as they do not represent the general adolescent female population.

### Interventions

Any girl-only PA programmes, defined for the purposes of this review as ‘organised, repeated PA activities, whether online, community, or school-based,’ were included. Multi-component programmes were eligible if PA was the primary focus. Only interventions lasting ≥ 4 weeks were included, based on the timeline required to see physical adaptations to exercise [28].

Using the SEAR framework [29], studies had to report either (i) the number Approached, and then Screened, Eligible, or Randomised or (ii) a pre-determined recruitment goal and the final number Screened, Eligible, or Randomised. For the purposes of this review, *Approached* was defined as the number of participants study authors reported as having been exposed to their recruitment strategies, *Screened* referred to participants assessed for eligibility after initial contact, *Eligible* referred to all those who met study inclusion criteria, and *Randomised* referred to those who were enrolled in the trial.

### Comparison

All studies were required to include a control group receiving either no PA programme, usual PE classes, or general PA advice.

### Outcomes

#### Primary outcome(s)

- Recruitment rate
- Percentage of recruitment goal
- Achievement of pre-determined recruitment goals
- Recruitment strategies employed

#### Secondary outcome(s)

- Challenges or facilitators to recruitment
- Mid-study changes to the recruitment process
- PA programme characteristics
- Retention rate

### Timeframe

No publication date limits were applied; studies published up to the original search date (3 February 2023) were included. The search was updated on 26 September 2024 to capture any new, relevant articles.

### Study type

This review included only individual and cluster RCTs, as RCTs are considered the ‘gold standard’ in effectiveness research, with higher standards regarding study conduct and reporting [30].

Unpublished, pre-print, non-peer-reviewed studies, conference abstracts, systematic reviews, narrative reviews, oral presentations not available in full text, and grey literature were excluded.

### Information sources and search strategy

Five electronic databases were searched: Embase, Medline, CINAHL, Web of Science, and the Cochrane Library – Central Trial Registry, alongside hand-search of citation lists. The search strategy included database-specific terms, truncations, and synonyms combining ‘adolescent females’, ‘recruitment’, and ‘physical activity’ (see Additional file 2 for full search strategy). Related publications were hand-searched for recruitment data.

### Data management

Studies were imported into EndNote 20.2 Desktop [31] and Covidence [32] for review management. Duplicates were removed automatically. After screening and selection, quantitative data was exported into IBM SPSS Statistics Version 28.0.1.0.

### Screening and selection

Title, abstract, and full-text screening were conducted independently by TOB in Covidence; two separate reviewers (EB, CD) completed the second author screening within Covidence. Discrepancies were resolved through team consensus discussions. Studies were excluded if required recruitment data were unavailable and original study authors did not respond after two contact attempts. For RCTs with multiple publications, data were collated under the most relevant parent study.

### Data extraction

A custom data extraction template including additional recruitment details was adapted from the Cochrane Review data extraction template [33]. Extracted data included study characteristics, participant demographics, intervention characteristics, and recruitment data (see Additional file 3 for complete list of data extracted).

One author (TOB) extracted all data. EB conducted duplicate extraction on 10% of studies; agreement was sufficient to proceed with single-author extraction. Discrepancies were resolved with a third reviewer.

### Data synthesis

This review focused on study recruitment methodologies, rather than pooled intervention effects, leading to two key methodological differences from traditional systematic reviews. Firstly, as discussed in the Prisma 2020 statement, meta-analysis is not an appropriate statistical method for all reviews [34]. In the current review, studies were not weighted by sample size but treated equally, using descriptive, correlational, and regression methods; analysis emphasised recruitment characteristics rather than pooled outcomes. Second, quality assessment was not applicable, as conventional tools assess risk of bias in intervention outcomes rather than recruitment processes and no established tool exists for evaluating recruitment methodology in this context. This approach is consistent with other health-related systematic reviews examining recruitment practices [35–38].

Due to the wide variety of PA types, these were condensed into logical categories via discussion by the research team. A literature search was undertaken to identify any preexisting recruitment category classification guidelines, and similar to a recent health-related recruitment review [38] recruitment strategies were categorised based on logical themes (see Additional file 3) based on the literature and discussion by the research team. Specifically, active and passive recruitment strategies were classified according to the degree of direct interaction with potential participants, consistent with definitions used in recruitment literature [39, 40]. In the broader literature, active recruitment typically involves direct interpersonal contact with the target population (e.g., telephone outreach or in-person presentations), whereas passive recruitment relies on indirect methods such as advertisements or mailings [41]. As most included studies were conducted in school settings, these definitions were operationalised using school-specific examples. *Active school-based* strategies included teacher-led recruitment of students, follow-up with absent students to obtain consent forms, and situations in which schools or teachers were financially supported to assist with the study. *Active researcher-led* strategies included researcher presentations to eligible students and the provision of PA taster sessions to all potentially eligible girls in the schools. Passive strategies included school-based flyers, information packs distributed through schools, and advertisements in school newsletters.

Recruitment conducted outside the school setting was categorised separately, as the setting in which recruitment occurred was considered likely to influence the processes and drivers of participation, and therefore required separate classification. These non-school-based strategies included social media advertising, researcher presentations to parents, mass mailings, and recruitment through external community locations such as churches or community organisations. This approach allowed classification to remain consistent with existing recruitment frameworks while reflecting the structural realities of school-based PA trials. Data analysis focused on three recruitment metrics:

1. Recruitment rate (participants randomised ÷ participants approached × 100%).
2. Percentage of pre-determined recruitment goal achieved (participants randomised ÷ pre-determined recruitment goal × 100%).
3. Met or did not meet 100% of pre-determined recruitment goal.

When calculating recruitment rate, where the number approached was not explicitly reported, the closest available denominator (most commonly the number screened or the number eligible) was used as a proxy for the number approached, as these values were considered the best available estimate of the population exposed to the recruitment strategy. This approach was used to allow calculation of recruitment rates while remaining as consistent as possible with the intent of the SEAR framework.

Retention rate (retained ÷ randomised × 100%) was analysed as a secondary outcome. Recruitment and retention analyses were stratified by setting (school-based vs. non-school-based). Subgroup comparisons for recruitment rate included SES (low, mixed, high), urban vs. rural, PA type, programme setting, recruitment strategies, and age group (11- < 15 vs. 15–19). Strategies used in successful vs. unsuccessful studies were compared.

Statistical analyses included Pearson’s correlations for continuous variables, point-biserial correlations for dichotomous variables, and Kendall’s Tau for ordinal variables. Correlations were examined between recruitment rate, percentage of pre-determined recruitment goal, achievement of recruitment goal (met vs not met), and retention rate, across demographic (e.g. SES, mean age), study design (e.g. feasibility versus fully powered), recruitment strategy, measurement approaches (e.g. accelerometer use), and PA programme (effectiveness, dosage, location, timing, type, and instructor) variables. Correlations were not run for subgroups with n < 10.

Normality of continuous variables was assessed using Shapiro–Wilk tests, histograms and PP plots; non-normal variables were log- or inverse-transformed prior to analysis. Significant correlations were followed by simple and multivariate linear regressions, with multicollinearity assessed using variance inflation factors (VIF). Model assumptions were checked using residual distributions, Durbin-Watson statistics, and PP plots. For binary outcomes, simple and multivariate logistic regressions were conducted, with model fit assessed using the Hosmer–Lemeshow test and odds ratios (Exp(B)) reported. Log-linearity of continuous predictors (e.g., mean age with binary recruitment outcomes) was evaluated using the Box-Tidwell test.

### Sensitivity analysis

A sensitivity analysis was conducted for the primary outcome, recruitment rate, excluding feasibility studies and retaining only fully powered studies. The purpose was to assess the robustness of the main findings and determine whether significant predictors of recruitment rate held when limiting the analysis to adequately powered studies. Descriptive statistics were not recalculated; instead, the analysis focused on regression models identified as significant in the all-studies analysis.

## Results

No deviations from the published protocol [25] were made throughout the systematic review process.

### Search and selection

**Fig. 1:**
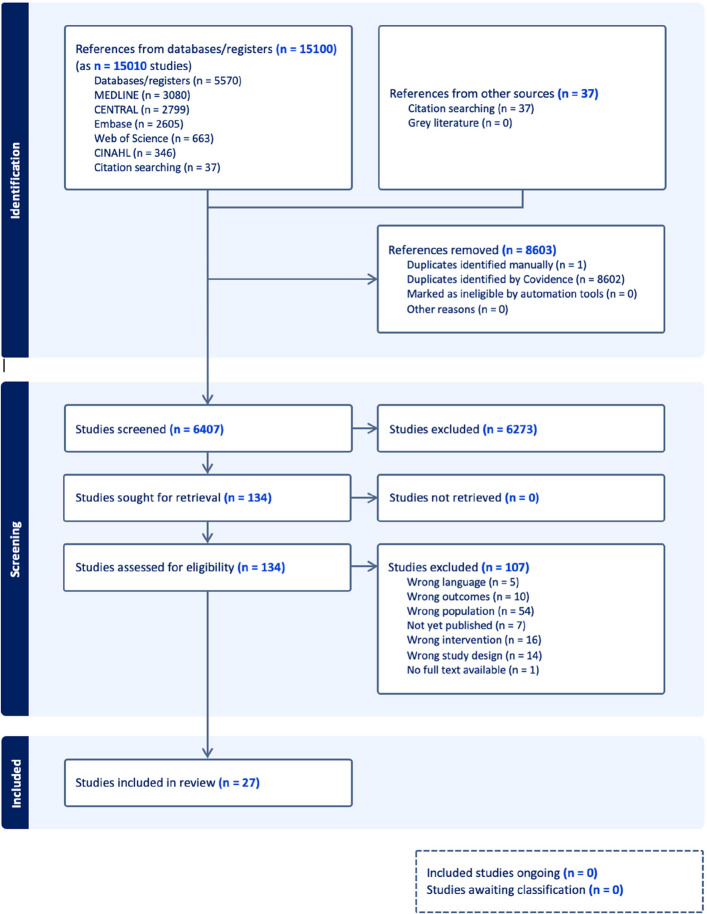
Preferred Reporting Items for Systematic Reviews and Meta-Analyses (PRISMA) diagram [22]

The search strategy retrieved 15,100 articles, with 37 of these found through citation hand-searches. After removing duplicates (*n* = 8,603), a total of 6,407 articles were screened by title and abstract. Following this, 6,273 studies were excluded, leaving 134 full-text articles. Before discussion for consensus, inter-rater reliability was high, with a proportionate agreement of 0.99 and a Cohen’s Kappa of 0.60. After consensus, 27 studies were included. See Fig. 1 for the PRISMA Diagram [22] for this review.

### Characteristics of included studies

Individual characteristics of included studies regarding year and country of publication, study design, demographics, and PA type are displayed in Table 1.

**Table 1:** Characteristics of individual studies

Study ID	PA Programme ID	School-based/non-school-based	Country	Mean Age (± SD)	SES**	Urban/Rural/Mixed	RCT Design	Study Design	PA Type
Neumark-Sztainer 2003 [42]	New Moves	SB	United States	15.8 (1.2)	Mixed	Urban	Cluster	Fully Powered	YD, FH, IA, TA
Stear 2003 [43]	Calcium exercise	SB	United Kingdom	17.3 (0.3)	-	-	Individual	Fully Powered	YD
Pate 2005 [44]	Lifestyle Education for Activity Program (LEAP)	SB	United States	13.6 (0.6)	Mixed	Mixed	Cluster	Fully Powered	W, YD, FH, IA
Schofield 2005 [45]	Pedometer	NSB	Australia	15.8 (0.8)	-	-	Cluster	Feasibility	W
Stevens 2005 [46]	Trial of Activity in Adolescent Girls (TAAG)	SB	United States	13.0 (0.1)	Mixed	Rural	Cluster	Fully Powered	YD, IA, TA
Young 2006 [47]	Life skills	SB	United States	13.8 (0.5)		Urban	Cluster	Fully Powered	TA
Dudley 2010 [48]	Linguistically diverse/Low SES	SB	Australia	16.5 (0.2)	Low	-	Individual	Feasibility	YD, TA
Lubans 2010 [49]	Nutrition and Enjoyable Activity for Teen girls (NEAT)	SB	Australia	13.2 (0.5)	Low	-	Cluster	Fully Powered	W, YD, FH, IA
Jago 2012 [50]	Bristol Girls Pilot	SB	United Kingdom	11–12*	-	Urban	Cluster	Feasibility	YD
Bernardoni 2014 [51]	Bone resistance training	SB	United States	11–12*	-	-	Cluster	Feasibility	FH
Casey 2014 [52]	Triple G Girls Get Going	SB	Australia	13.4 (0.9)	Low	Rural	Cluster	Fully Powered	TA
Kahlin 2014 [53]	Swedish inactive girls	NSB	Sweden	16.7 (0.5)	-	-	Cluster	Fully Powered	FH
Nogueira 2014 [54]	Capoeira (CAPO) Kids	SB	Australia	10.6 (0.6)	-	-	Cluster	Fully Powered	FH, IA
Jago 2015 [55]	Bristol Girls RCT	SB	United Kingdom	11–12*	Mixed	Urban	Cluster	Fully Powered	YD
Leme 2015 [56]	Healthy Habits Healthy Girls	SB	Brazil	16.1 (0.1)	Low	Urban	Cluster	Fully Powered	W, YD, FH, IA
Lander 2017 [57]	Early adolescent motor skills	SB	Australia	12.5 (0.3)	-	-	Cluster	Feasibility	FH, IA, TA
Okely 2017 [58]	Girls in Sport	SB	Australia	13.6 (0.0)	Mixed	Mixed	Cluster	Fully Powered	W, YD, IA, TA
Carlin 2018 [59]	Walking In ScHools (WISH) pilot	SB	United Kingdom	12.6 (0.5)	-	-	Cluster	Fully Powered	W
Linck 2018 [60]	Step it up with Fitbit Zip	NSB	United States	16.6 (1.2)	-	Urban	Individual	Feasibility	W
Robbins 2019 [61]	Girls on the Move	SB	United States	12.1 (1.1)	Low	Urban	Cluster	Fully Powered	YD, FH, TA
Latino 2019 [62]	Multilateral training	SB	Italy	14.4 (0.5)	-	-	Individual	Fully Powered	W, FH, IA, TA
Dunker 2021 [63]	Brazilian New Moves	SB	Brazil	13.4 (0.6)	Low	-	Cluster	Fully Powered	YD, FH, IA, TA
Kim 2021 [64]	Psychological modification-based PA	-	South Korea	14.4 (0.7)	-	Urban	Individual	Fully Powered	FH, IA
Cowley 2022 [65]	HERizon project	NSB	United Kingdom & Ireland	14.9 (1.2)	Mixed	Mixed	Cluster	Fully Powered	YD, FH, IA
White 2022 [66]	Health and Well-Being for Girls (HWBG)	SB	Australia	14.0 (0.5)	Low	-	Cluster	Feasibility	W, YD, FH, TA
Murphy 2023 [67]	WISH RCT	SB	United Kingdom & Ireland	12–14*	Mixed	Mixed	Cluster	Fully Powered	W
Pike 2023 [68]	Goals for Girls	SB	South Africa	15.3 (1.5)	Low	Urban	Cluster	Fully Powered	YD, TA

*SB* school-based, *NSB* non-school-based,— not reported

*W *walking,* YD *yoga or dance,* FH *fitness or High Intensity Interval Training (HIIT),* IA *individual activities,* TA *team activities

* = authors did not report participant mean age, only age range of participants

**SES: socioeconomic status

Most (84.6%, *n* = 22/26; 1 not reported) programmes were school-based, with 63.6% (*n* = 14) of these replacing timetabled physical education (PE) classes [42, 44, 46–48, 51, 52, 54, 56–58, 62, 66, 68] and 59.1% (*n* = 13) offering PA opportunities outside of class time [43, 44, 46, 49, 50, 55, 58, 59, 61, 63, 67, 68], either before school [46, 59, 67], during lunch [43, 46, 49, 56, 58, 59, 67], or directly after school [43, 44, 46, 49, 50, 55, 58, 61, 63, 68]. The remaining 15.4% (*n* = 4) of non-school-based programmes [45, 53, 60, 65] were offered through local community PA centres (3.8%, *n* = 1) [53], online (3.8%, *n* = 1) [65], or remotely using activity trackers (11.5%, *n* = 3) [45, 60, 65]. It should be noted that these percentages do not sum to 100% as most programmes offered more than one PA option; for example, several school-based programmes both replaced PE class and offered before school/during lunch/after-school class options [44, 46, 56, 58, 68]. In the case where programmes combined both school-based and non-school-based options [46, 49, 52, 58], programmes were classified as school-based as the students were recruited from schools.

Most programmes were delivered by teachers (51.9%, *n* = 14) [42, 44, 46–49, 51, 52, 54, 56–58, 63, 64, 66] or community fitness instructors (40.7%, *n* = 11) [42, 44, 46, 50, 52, 53, 55, 58, 61, 63, 65]; others included members of a research team (18.5%, *n* = 5) [43, 45, 60, 65, 66], peers (14.8%, *n* = 4) [49, 56, 59, 67], near-peers (3.7%, *n* = 1) [68], university PE graduates (3.7%, *n* = 1) [62], or researchers who were also teachers at the relevant schools (3.7%, *n* = 1) [66]. The mean age of study participants was 14.1 years (SD 1.8), ranging from 10.6–17.3 years.

A total of 77.8% (*n* = 21) of PA interventions were designed based on underpinning behaviour change theories [42, 44, 46, 48–50, 52, 53, 55–61, 63–68], such as Self-Determination Theory [69]. Of the 81.5% (*n* = 22) of included studies that aimed to increase PA levels in adolescent girls [42, 44–50, 52–56, 58–61, 63–67], only 40.9% (*n* = 9) were successful at increasing PA [44–46, 53, 54, 56, 59, 64, 66].

### Recruitment and retention rates

See Table 2 for overall mean and median recruitment rates, recruitment rate by subgroup, percentage of pre-determined recruitment goal achieved, and retention rates. Only five of the 27 studies [47, 48, 53, 70, 71] reported all four SEAR (Screened, Eligible, Approached, Randomised) framework [72] elements. Of the 27 included studies, 18 provided a recruitment denominator that permitted the calculation of a recruitment rate (see Table 2). Of these 18, however, five [49, 51, 54, 63, 65] did not explicitly state the number ‘approached’, with authors instead reporting only the number screened or the number eligible. As a result, a proxy denominator (the number screened or eligible) was used to calculate recruitment rate, as per Methods.

**Table 2:** Mean and median overall recruitment rates, recruitment rates by subgroup, and percentage of pre-determined recruitment goals

Category	n	Mean	Median	± SD	Skew	Std Err Skew	Range	Min	Max
Overall mean and median recruitment rates (%)									
All studies^1^	18	62.6*	60.5	27.8	.01	.5	75.7	23.6	99.3
School-based studies^2^	15	59.3*	50.0	28.4	.3	.6	75.7	23.6	99.3
Non-school-based studies^3^	3	78.9	86.0*	20.1	−1.4	1.2	38.2	56.2	94.4
Recruitment rates (%) by socioeconomic status									
Low SES	5	56.5*	36.8	35.0	.6	.9	75.6	23.6	99.2
Mixed SES	5	63.2*	80.0	28.6	-.6	.9	55.6	30.4	86.0
High SES	0	N/A	N/A	N/A	N/A	N/A	N/A	N/A	N/A
Recruitment rates (%) by urban versus rural location									
Urban	4	39.3*	38.5	8.2	-.6	1.0	19.6	30.4	50.0
Rural	2	**56.9**	56.9	32.7	N/A	N/A	46.3	33.7	80.0
Mixed	3	68.5	85.8*	30.2	−1.7	1.2	54.4	33.6	86.0
Recruitment rates (%) by younger versus older adolescent age									
Mean age 10- < 15	14	65.9*	72.4	26.7	-.1	.6	66.2	33.2	99.3
Mean age 15–19	4	51.2	43.3*	32.1	1.0	1.0	70.8	23.6	94.4
Recruitment rates (%) for school-based studies: replace PE versus before/during lunch/after school									
Replace PE	9	62.3*	64.7	28.1	-.1	.72	75.7	23.6	99.3
Before/during lunch/after school	6	54.9*	38.5	30.9	1.0	.9	68.8	30.4	99.2
Recruitment rates (%) by PA type									
Walking	5	69.2*	85.8	33.1	-.5	.9	66.0	33.2	99.2
Yoga/dance	10	60.5*	60.1	29.8	.0	.7	75.6	23.6	99.2
Fitness/HIIT	9	72.8*	86.0	25.8	-.6	.7	65.7	33.6	99.3
Individual activities	8	82.9	87.7*	21.0	−2.3	.8	65.7	33.6	99.3
Team activities	8	61.2*	65.0	28.0	-.2	.8	66.5	23.6	90.1
Recruitment rates (%) by recruitment strategies									
Incentives	4	51.2	43.3*	32.1	1.0	1.0	70.8	23.6	94.4
Active school	5	69.7*	80.0	27.0	-.5	.91	65.5	33.7	99.2
Active researchers	8	52.1*	45.1	22.0	.9	.8	59.0	30.4	89.4
Passive school	9	54.7*	40.1	25.6	.6	.7	59.7	30.4	90.1
Online/community	2	68.0	**68.0**	25.5	N/A	N/A	36.0	50.0	86.0
Overall mean and median % of pre-determined recruitment goals									
All studies^1^	19	115.9	104.0*	33.9	1.2	.5	161.9	48.9	210.8
School-based studies^2^	15	122.5*	107.0	33.0	1.7	.6	115.7	95.1	210.8
Non-school-based studies^3^	4	91.3	102.6*	28.6	−1.9	1.0	62.2	48.9	111.1

^**1**^total *n* = 27; ^**2**^ total *n* = 22; ^**3**^ total *n* = 4

^*^The mean was bolded when the skew was low (between -.05 and 0.5) or moderate (between -.05 and −1 or 0.5 and 1), indicating a more normal distribution, and the median was bolded when skew was high (below −1 or above 1), indicating that the median was a better measurement of central tendency in this case

Only 19 (70.4%) studies reported whether predetermined recruitment goals were met, and of these 31.6% (*n* = 6) failed to do so. School-based studies were less likely to meet their pre-determined recruitment goal as compared to non-school-based studies (66.7%, *n* = 10 successful versus 75.0%, *n* = 3). Subgroup analysis based on the recruitment strategies employed and the likelihood of meeting or not meeting the recruitment goal was also performed; successful programmes were more likely to have had both the school (30.8% in successful programmes versus 16.7% in unsuccessful programmes) and the research team (53.8% versus 16.7%) actively involved in the recruitment process, such as by having teachers assist in organising and promoting the programme and/or researchers presenting directly to eligible participants about the study.

The retention rate to the studies was high, with a mean of 84.9% (± 13.25, *n* = 22). School-based studies experienced higher retention (86.0% ± 12.6, *n* = 17) than non-school-based studies (81.2% ± 16.3, *n* = 4).

### Recruitment strategies

A total of 77.8% (*n* = 21) of studies reported on recruitment strategies employed (see Table 3 for the recruitment strategies employed in individual studies, alongside their recruitment and retention data).

**Table 3:** Individual study recruitment strategies employed and recruitment data including recruitment rate, percentage of recruitment goal achieved, whether they were successful at meeting their pre-determined recruitment goal, and whether the authors noted recruitment challenges

	Recruitment strategies					Recruitment data				Retention data	
Study ID	**Incentives**	**Active school**	**Active researchers**	**Passive school**	**Online community**	**Recruitment rate (%)**	Percentage (%) of recruitment goal achieved*	**Met pre-determined recruitment goal**	**Authors noted recruitment challenges**	**Retention rate (%)**	**Authors noted attendance challenges**
Neumark-Sztainer 2003 [42]	x	x	x	✓	x	-	98.9	x	No	94.1	No
Stear 2003 [43]	-	-	-	-	-	-	120.0	✓	No	91.0	Yes
Pate 2005 [44]	✓	x	✓	x	x	33.6	-	-	No	76.9	No
Schofield 2005 [45]	-	-	-	-	-	94.4	-	-	No	80.0	No
Stevens 2005 [46]	✓	✓	✓	x	x	80.0	107.0	✓	Yes	-	No
Young 2006 [47]	-	✓	✓	x	✓	50.0	-	-	Yes	95.0	No
Dudley 2010 [48]]	-	-	-	-	-	23.6	-	-	Yes	68.4	No
Lubans 2010 [49]	✓	✓	x	x	x	99.2	99.2	x	No	82.0	No
Jago 2012 [50]	✓	x	✓	✓	x	40.1	-	-	No	-	Yes
Bernardoni 2014 [51]	x	x	x	x	x	64.7	157.1	✓	No	100.0	No
Casey 2014 [52]	x	✓	x	✓	x	33.7	-	-	Yes	72.1	No
Kahlin 2014 [53]	x	x	✓	✓	x	56.2	104.0	✓	No	90.4	Yes
Nogueira 2014 [54]	-	-	-	-	-	99.3	-	-	No	91.4	No
Jago 2015 [55]	✓	x	✓	✓	x	30.4	126.9	✓	Yes	-	Yes
Leme 2015 [56]	x	x	✓	x	x	-	95.1	x	No	75.1	No
Lander 2017 [57]	x	x	x	✓	x	90.1	-	-	No	95.0	No
Okely 2017 [58]	✓	✓	x	✓	x	85.8	210.8	✓	No	81.8	No
Carlin 2018 [59]	✓	x	x	✓	x	33.2	99.5	x	No	99.0	No
Linck 2018 [60]	x	x	x	✓	✓	-	48.9	x	Yes	79.6	No
Robbins 2019 [61]	✓	x	✓	✓	x	36.8	103.7	✓	No	58.2	Yes
Latino 2019 [62]	X	x	x	✓	x	-	166.7	✓	No	100.0	No
Dunker 2021 [63]	X	x	✓	x	x	89.4	116.4	✓	No	-	Yes
Kim 2021 [64]	-	-	-	-	-	-	111.1	✓	No	100.0	No
Cowley 2022 [65]	x	x	x	✓	✓	86.0	101.3	✓	Yes	56.2	No
White 2022 [66]	-	✓	✓	✓	x	-	100.0	✓	No	98.9	No
Murphy 2023 [67]	✓	✓	✓	✓	x	-	136.3	✓	No	-	Yes
Pike 2023 [68]	✓	x	x	x	x	-	99.7	x	Yes	82.5	Yes

✓ = yes; x = no;— = data not reported

^*^Percentages may reach greater than 100% if actual number of participants randomised was greater than the pre-determined recruitment goal

Passive recruitment strategies in schools, such as distributing information packages to all eligible participants, were the most common recruitment strategy category (66.7%; *n* = 14), followed by researchers actively recruiting at schools, such as by presenting about the study to eligible participants (52.5%; *n* = 11) and the use of incentives (47.6%; *n* = 10). The least common recruitment strategy categories were teachers/schools actively recruiting (33.3%; *n* = 7), and recruitment performed online or out in the community (14.3%, *n* = 3). Studies that reported on recruitment strategies typically described a bundle, or combination, of strategies employed and did not distinguish or report on the effectiveness of individual recruitment strategies.

Overall, 29.6% (*n* = 8) of study authors reported challenges with recruitment. Of these, 75.0% (*n* = 6) were school-based. Similarly, 29.6% (*n* = 8) of authors reported challenges with attendance, with 87.5% (*n* = 7) of these being school-based. Overall, only two studies [55, 68] reported challenges with both recruitment and attendance.

### Effectiveness of recruitment strategies and programme design factors

Recruitment strategies within the included trials were not themselves randomised, or this was not reported, and as noted above, they were usually implemented in bundled combinations rather than as isolated components. Consequently, it was not possible to determine the true effectiveness of specific recruitment approaches, and any observed associations between individual strategies and recruitment outcomes should be interpreted as descriptive and hypothesis-generating rather than causal. While all recruitment strategy condensed categories (e.g. active school, incentives) and individual recruitment strategies (e.g. flyers, videos, researcher presentations) were run in correlation analyses against recruitment rate, none showed statistically significant associations with recruitment rate. Whereas programme design factors such as offering the programme remotely, providing individual activities, or the study aiming to improve participants’ Body Mass Index (BMI) were significant (see Table 4). Correlations were not performed for non-school-based studies due to the small sample size (*n* = 4).

**Table 4:** Significant regression results for recruitment rate and percentage of pre-determined recruitment goal, correlation results for meeting/not meeting pre-determined recruitment goal, and regression results for retention rate

Recruitment rate significant simple linear regression results									
Outcome (dependent variable)	Predictor (independent variable)				±	R^2^	F	Df regression, residual	p
	All studies								
Recruitment rate	Programme offered online or remotely (with activity trackers)				+	.259	5.6	1, 16	.031*
	Individual activities				+	.457	13.5	1, 16	.002**
	Aim to improve BMI				+	.308	7.1	1, 16	.017*
	School-based studies								
	Individual activities				+	.624	21.6	1, 13	<.001**
	Aim to improve BMI				+	.447	10.5	1, 13	.006**
	Teacher instructor				+	.283	5.1	1, 13	.041*

Recruitment rate significant multivariate linear regression results									
Dependent variable	Independent variables	B	Std. Error	t	R^2^	F	Df Regression, Residual	p	VIF
	All Studies								
Recruitment rate	IV1 Programme offered online or remotely IV2 Aim to Improve BMI	51.9	5.8	8.9	.473	6.7	2, 15	.008**	
		IV1 29.9	IV1 13.8	IV1 2.2				IV1.047*	1.0
		IV2 34.1	IV2 13.8	IV2 2.5				IV2.026*	1.0
Recruitment rate	IV1 Programme offered online or remotely IV2 Individual Activities	43.5	6.0	7.3	.602	11.4	2, 15	<.001**	
		IV1 28.1	IV1 12.0	IV1 2.3				IV1.033*	1.0
		IV2 32.4	IV2 9.0	IV2 3.6				IV2.003**	1.0

Percentage of pre-determined recruitment goals significant linear regression results									
Outcome (dependent variable)	Predictor (independent variable)				±	R^2^	F	Df regression, residual	p
	All studies								
Percentage of recruitment goal	Programme offered on school property				+	.254	5.5	1, 16	.033*
	Programme offered online or remotely				-	.279	6.2	1, 16	.024*
	Soccer				+	.221	4.8	1, 17	.042*
	Basketball				+	.256	5.9	1, 17	.027*
	Tennis				+	.278	6.6	1, 17	.020*
	Aim to reduce depression				-	.465	14.8	1, 17	.001**
	Schools/teachers paid				+	.322	6.7	1, 14	.022*
	Presentation to parents				-	.483	13.1	1, 14	.003**
	School-based studies								
	Basketball				+	.304	5.7	1, 13	.033*
	Tennis				+	.357	7.2	1, 13	.019*
	Aim to decrease sedentary time				+	.265	4.7	1, 13	.049*
	Schools/teachers paid				+	.458	9.3	1, 11	.011*

Percentage of pre-determined recruitment goals significant multivariate linear regression results									
Dependent variable	Independent variables	B	Std. Error	t	R^2^	F	Df Regression, Residual	p	VIF
	All Studies								
Percentage of recruitment goal	IV1 Presentation to parents IV2 Schools/teachers paid	2.0	.0	99.7	.735	18.1	2, 13	<.001**	
		IV1 -.3	IV1.1	IV1 −4.5				IV1 <.001**	1.0
		IV2.2	IV2.1	IV2 3.5				IV2.004**	1.0
Percentage of recruitment goal	IV1 Schools/teachers paid IV2 Programme offered on school property	1.9	.1	32.0	.479	6.0	2, 13	.014*	
		IV1.2	IV1.1	IV1 2.4				IV1.030*	1.0
		IV2.1	IV2,1	IV2 2.0				IV2.070*	1.0

Retention rate significant linear regression results									
Outcome (dependent variable)	Predictor (independent variable)				±	R^2^	F	Df regression, residual	p
	All studies								
Retention Rate	Yoga/dance/pilates/Zumba/aerobics				-	.232	6.0	1, 20	.023*
	Fitness or FMS assessments				+	.243	6.4	1, 20	.020*
	Dance				-	.403	13.5	1, 20	.002**
	Delivered by Community Fitness Instructors				-	.221	5.4	1, 19	.031*
	Paid advertising on Instagram				-	.283	5.5	1, 14	.034*
	School-based studies								
	Yoga/Dance/Pilates/Zumba/Aerobics				-	.246	4.9	1, 15	.043*
	Community Fitness Instructors				-	.246	4.9	1, 15	.043*
	Recruitment Video				-	.409	7.6	1, 11	.019*
	Study Clothing				-	.409	7.6	1, 11	.019*
	Gifts for participation				-	.385	6.9	1, 11	.024*
	Paid for Consent form return				-	.409	7.6	1, 11	.019*

+ positive direction of association;—negative direction of association

*Results significant at *p* < 0.05

**Results significant at p < 0.01

Shapiro–Wilk tests indicated that the variable *percentage of recruitment* goal violated normality in the all-studies analysis; it was therefore log-transformed by 10 before correlation analysis. In school-based studies for this variable, log transformation was insufficient to achieve normality; therefore, the variable was inverse-transformed. The variable *mean age* satisfied the assumption of log-linearity in relation to the binary outcome of meeting or not meeting recruitment goals, as confirmed by a non-significant Box-Tidwell interaction (*p* = 0.740). Mean age was therefore retained as a continuous predictor in logistic regression models.

Significant correlations for the continuous outcomes of recruitment rate, percentage of recruitment goal, and retention rate are presented in Additional files Item 4, with significant regression results below in Table 4. Multivariate regression was performed between the relevant retention rate linear regression findings, but none were significant.

Significant correlations with the binary variable of meeting the pre-determined recruitment goal were not significant when carried forward into simple and multivariate logistic regression; therefore, the correlation results are presented below.

### Meeting or not meeting pre-determined recruitment goals

Across all studies, meeting recruitment goals was more likely when researchers were actively involved (e.g., school presentations, taster sessions) (r(14) = 0.516, *p* = 0.041), and less likely when peer instructors delivered these sessions (r(16) = 0.472, *p* = 0.048). In school-based trials, peer instruction remained negatively associated with meeting recruitment goals (r(13) = 0.533, *p* = 0.041). Studies’ aiming to improve participants’ BMI was negatively correlated with the likelihood of meeting pre-determined recruitment goals (r(13) = 0.533, *p* = 0.041).

### Sensitivity analysis

A sensitivity analysis was conducted for our primary outcome, recruitment rate, including only fully powered studies. In this reduced sample, programme delivery online or remotely (with activity trackers) was no longer significant. Offering individual activities, however, still significantly predicted higher recruitment rates (R^2^ = 0.575, F(1, 10) = 3.6, *p* = 0.004) and an explicit aim to improve participants’ BMI also remained significant (R^2^ = 0.461, F(1, 10) = 8.6, *p* = 0.015).

### Narrative reporting of barriers, facilitators, and changes to recruitment strategies mid-study

Only two studies [46, 68] reported adapting their recruitment strategies mid-study. One study [68] improved engagement by allowing schools to run sessions either during or after-school and increased WhatsApp reminders.

Another [46] employed an iterative approach introducing (1) staggering their recruitment timelines into sequential blocks to allow for more focused attention from staff at each school; (2) paying teachers to help organise and follow up with adolescent participants; (3) employing small group recruitment presentations rather than whole-school; (4) offering gift certificates to participants as incentives to complete all outcome measures; (5) reducing the length of the consent form and using simpler terms at school visits; and (6) standardising recruitment staff’s approach to ensure they were friendly and engaging.

## Discussion

Recruitment of adolescent girls remains a persistent challenge for PA programme providers [8, 12–14], with relatively little research examining how best to recruit this demographic. This quantitative review complements existing qualitative work exploring barriers, facilitators, and perceptions of PA among adolescent girls [17, 19, 73, 74]. Interpretation of the findings, however, was limited by incomplete reporting of recruitment procedures and by differences in how recruitment denominators were defined. Recruitment rates could only be calculated for a subset of included trials, and most of these were school-based, meaning that the conclusions relate primarily to recruitment conducted within school systems. All quantitative comparisons in this review should be interpreted as descriptive and hypothesis-generating rather than confirmatory, given the small study sample, predominantly school-based evidence, and heterogeneous/limited reporting that reduces comparability and precision. These findings indicate associations that warrant further testing rather than confirmatory relationships; replication in non-school-based community and digital programme contexts is essential to validate these patterns.

### Denominator definition and reporting of recruitment stages

A central issue across the literature examining recruitment was the inconsistent definition of the population used to calculate recruitment rates. In recruitment research, it is useful to distinguish between several stages: the total eligible population within a school or community (the “grand denominator”), the proportion exposed to recruitment activities, the proportion who respond or enter screening, and the proportion who ultimately enrol or are randomised [41]. Reporting these stages allows recruitment to be interpreted in terms of both reach and conversion through the recruitment process. Recruitment rates derived from poorly defined or inconsistently operationalised denominators are not directly comparable across studies or settings.

In the present review, recruitment data was gathered using the SEAR framework (Screened, Eligible, Approached, Randomised) [72]; however, most included studies collapsed or did not report all stages of recruitment, and the information required to complete the full sequence was frequently missing. Moreover, the number reported as “approached” may have in reality represented the entire eligible cohort (e.g., all girls in a year group), with no clear indication of how many participants were actually exposed to the recruitment strategy. It was often unclear whether eligible participants received recruitment materials, attended presentations, or were present when recruitment was introduced, making the true reach of the strategies impossible to determine. Even using the broader definition of “approached” adopted in this review, which allowed inclusion provided that the number of participants exposed to recruitment strategies was reported (even if this corresponded to the number eligible or the number who responded; see *Non-school-based studies* subsection), recruitment rates could only be calculated for 18 of the 27 included studies. Differences in recruitment rates may therefore reflect variation in reporting rather than true differences in recruitment effectiveness, and all quantitative comparisons should be interpreted as hypothesis-generating only, rather than evidence that any recruitment strategy is superior.

### Recruitment rates for adolescent girls into PA RCTs

The mean recruitment rate across RCTs was 62.6% (± 27.8). Compared with similar systematic reviews of recruitment rates, adolescent girls were harder to recruit into PA RCTs than adults into PA programmes in the workplace [75], exercise therapy with multimorbidity [76], or all-topic health-related RCTs [77], but easier to recruit than adolescents into mental health [78] or weight management [79] trials.

Recruitment rates were higher in younger (age 11- < 15; 65.9% ± 28.1) versus older (age 15–19; 51.2% ± 32.1) girls, reflecting the well-documented declines of PA participation with age [80], perhaps due to shifting interests, social dynamics, and competing priorities such as academic pressures [17, 81].

### Impact of socioeconomic status

Subgroup analyses revealed that it is harder to recruit low SES adolescent girls (recruitment rate 56.5% ± 35.0), as compared with girls from mixed SES backgrounds (63.2% ± 28.6), echoing well-established evidence that socioeconomic disparities strongly influence PA participation [82]. Girls from lower-income households are consistently less active and face more barriers to participation [83] such as cost, transport, safety, and access to facilities [84], which makes recruitment particularly challenging in disadvantaged communities. Moreover, only 2/27 studies [63, 68] were published in the Global South, highlighting a major geographic imbalance in the literature. The absence of high-quality adolescent PA RCTs from low- and middle-income countries (LMIC) is concerning given that adolescent girls in low-SES settings experience disproportionately high inactivity levels [21, 84]. Strengthening the evidence base in LMIC contexts is therefore critical for designing scalable and equitable PA interventions that address global disparities.

### School-based studies

School-based studies had slightly lower mean recruitment (59.3% ± 8.4), particularly when programmes were offered before, during lunch, or after school (54.9% ± 30.9), when attendance was optional. It should be noted that recruitment into PA programmes and recruitment into PA research studies are distinct entities. Programmes replacing PE classes likely had higher actual participation rates than recorded, as participation was mandatory but parental consent was required for inclusion in studies. Recruitment varied widely (23.6–99.2%), and inconsistent reporting means these results should be interpreted cautiously.

### Non-school-based studies

Of the 18 studies for which a recruitment rate could be calculated, 15 were school-based and only 3 were conducted outside the school setting, limiting the strength of any conclusions regarding the relative effectiveness of recruitment strategies in different contexts. Moreover, non-school-based studies often relied on social media advertisements or voluntary sign-up, where the total number of individuals exposed to recruitment (or the recruitment denominator) was not reported. In these studies, the number reported as “approached” may include only those who actively responded, which can artificially inflate calculated recruitment rates. In an online intervention [85], for example, recruitment was conducted through social media advertising, but the number of adolescents who were exposed to the advertisements was not reported. The recruitment denominator therefore reflected only those who contacted the research team after seeing the recruitment materials, making it impossible to determine the true reach of the strategies. Consequently, although non-school-based programmes appeared to show higher recruitment rates, these findings were highly sensitive to denominator differences and may have been artificially inflated. Furthermore, these non-school-based findings were based on only three studies, limiting power and true comparisons with school-based studies. The apparent advantage of non-school recruitment should be interpreted cautiously and not viewed as conclusive evidence that non-school settings are more effective for recruiting adolescent girls into PA programmes.

### Associations between recruitment strategies and recruitment outcomes: teachers and researchers mattered

No traditional recruitment strategies (e.g. flyers, videos) showed significant correlations with recruitment rate. Teacher and school involvement, however, consistently emerged as important. This finding was repeatedly reflected in our results: (1) RCTs that employed active participation from teachers and schools in the recruitment process observed the highest mean recruitment rates; (2) recruitment rates were significantly improved when teachers were the PA instructors; (3) the percentage of recruitment goal achieved was significantly higher in studies where schools or teachers were paid to help organise and recruit to the programme; and (4) studies that successfully met their pre-determined recruitment goals were more likely to have had schools actively involved in the recruitment process. Our results echo wider findings that schools, with their structured programmes, supportive settings [86], and daily student attendance [87], are ideal for PA promotion, and recruitment improves with personal relationships [88] and community partnerships with known and trusted link persons [89]. Researcher involvement also mattered: school presentations and “try-it” taster sessions were positively correlated with meeting recruitment goals.

### Incentives

Incentives targeting the adolescent girls themselves were associated with the lowest recruitment rates (mean 51.2%, median 43.3% ± 32.1) and significantly lower retention in school-based studies (gifts for participation R^2^ = 0.385, F(1,11) = 6.9, *p* = 0.024; study clothing gift R^2^ = 0.409, F(1, 11) = 7.6, *p* = 0.019; paid consent form return R^2^ = 0.409, F(1,11) = 7.6, *p* = 0.019), suggesting that extrinsic motivators may be associated with lower intrinsic engagement with PA. The percentage of recruitment goal achieved, however, was significantly higher in both all studies (R^2^ = 0.322, F(1, 14) = 6.7, *p* = 0.022) and school-based studies (R^2^ = 0.458, F(1, 11) = 9.3, *p* = 0.011) where schools or teachers were financially incentivised to help organise and recruit to the programme. This suggests that, so long as ethical considerations such as avoiding undue pressure on adolescent participants can be navigated, allocating financial resources to support teacher involvement, rather than adolescent girls, may be recommended.

### PA programme design factors

Online or remote delivery (with activity trackers) was associated with higher recruitment in the all-studies analysis (R^2^ = 0.259, F(1, 16) = 5.6, *p* = 0.031), reflecting adolescents’ interest in flexible, technology-supported PA. Interventions that included the aim to reduce depression, however, recruited lower percentages of their recruitment goal (R^2^ = 0.465, F(1, 17) = 14.8, *p* = 0.001), highlighting the inherent challenge of engaging individuals with depression in PA, despite robust evidence supporting its benefits [90]. BMI-focused programmes showed mixed associations: positively linked with recruitment rate (R^2^ = 0.308, F(1, 16) = 7.1, *p* = 0.017) but negatively correlated with meeting recruitment goals in school-based studies (r(13) = 0.533, *p* = 0.041). These inconsistencies highlight the need for more nuanced exploration of weight-focused framing in recruitment, but should also be treated with caution, as our sample size was limited.

PA type preferences varied: individual activities (e.g., running, skipping, martial arts) were positively correlated with recruitment rates (R^2^ = 0.457, F(1, 16) = 13.5, *p* = 0.002), while team sports (e.g., soccer R^2^ = 0.221, F(1, 17) = 4.8, *p* = 0.042, basketball R^2^ = 0.256, F(1, 17) = 5.9, *p* = 0.027) were associated with higher percentages of recruitment goal achieved. These results mirror prior research [91] which found that while adult PA preferences demonstrated a consistent pattern of participation, child and adolescent preferences were highly variable across contexts, reinforcing the need for tailoring PA offerings to local microcultures.

### Retention rates for adolescent girls in PA RCTs

Retention was a secondary focus; therefore, PA RCTs reporting retention but lacking quantitative recruitment data were excluded. While our findings provide useful insights, they should not be viewed as definitive review of retention rates.

Retention (84.9% ± 13.3) exceeded recruitment, with higher retention in school-based (86.0% ± 12.6) versus non-school-based programmes (81.2% ± 16.3), echoing similar findings from adult PA studies conducted at workplaces [75] and emphasising that the convenience of programme timing and location is important for retention. Fitness testing also improved retention (R^2^ = 0.243, F(1, 20) = 6.4, *p* = 0.020), possibly because girls valued monitoring progress and developing mastery. Dance-based interventions were associated with lower retention (R^2^ = 0.403, F(1, 20) = 13.5, *p* = 0.002), highlighting the importance of further exploring how activity type shapes continued engagement.

### Sensitivity analysis

Excluding feasibility studies and restricting to fully powered trials produced mixed results. Online or remote delivery was no longer significant, while offering individual activities remained strongly associated with higher recruitment (R^2^ = 0.575, F(1, 10) = 13.6, *p* = 0.004). An explicit aim to improve BMI was also significant (R^2^ = 0.461, F(1, 10) = 8.6, *p* = 0.015). These findings suggest that feasibility and fully powered studies may operate under different recruitment dynamics. While feasibility studies may have smaller sample sizes and, therefore, potentially more intensive researcher involvement, fully powered trials may have greater resources to dedicate to recruitment. Caution is warranted, however, as the smaller sample in our analysis reduced power. Future reviews should stratify feasibility and fully powered studies to clarify whether recruitment strategies function differently by study type. Moreover, while schools provide convenient access to large cohorts of adolescent girls, the reliance on this setting constrains transferability to real-world, voluntary PA contexts such as community clubs or online initiatives. Broadening recruitment research beyond educational settings will be key to advancing equitable PA promotion.

### Limitations

Consistent with prior reviews [77, 88, 92, 93], recruitment data were inconsistently reported. Nearly 30% of studies did not state recruitment goals, and one-third did not report how many participants were approached, constraining precision and limiting statistical power. Ten otherwise eligible studies were excluded due to lack of quantitative recruitment data [94–103], which may bias the evidence towards better reported studies.

The included literature was predominantly school-based, limiting generalisability to other settings (e.g. community or online-based programmes) where gatekeepers, consent procedures, and incentive structures may differ. Reporting of recruitment strategies was often sparse or non-standardised (e.g. differing definitions of the denominator “approached,” incomplete descriptions of recruitment strategies), which reduced comparability across studies.

Given the small number of eligible studies, heterogeneous measurement and definitions, and frequent co-occurrence of strategies (recruitment strategies are not employed in isolation, which complicates the isolation of individual effects), these regression analyses should be interpreted as exploratory and hypothesis-generating rather than confirmatory. Moreover, the recruitment strategies examined in this review were not themselves randomised, and similar to findings from a recent scoping review examining recruitment strategies reported in health promotion and disease prevention RCTs [104], they were commonly implemented in bundled combinations rather than as isolated components. This limits causal interpretation: observed associations between a recruitment strategy (e.g., teacher involvement, presentations, incentives) and recruitment outcomes may reflect confounding by setting, study design, resourcing, or denominator definition rather than a true strategy effect. This further strengthens the above comment that associations should be considered descriptive rather than causal in nature.

Lastly, while only 68.4% (*n* = 13) of studies achieved 100% of recruitment goals, most reached 95–98%. As recruitment targets are often inflated above the minimum power requirement, some studies may have ceased recruitment once the minimum numbers were met. Thus, a binary success/failure classification may not accurately reflect recruitment effectiveness. Standardised benchmarks and improved reporting are needed to facilitate clearer comparisons, support future reviews, and facilitate evidence-based recommendations for recruitment strategies targeting adolescent girls.

## Conclusion

This systematic review identifies recruitment strategies associated with improved enrolment of adolescent girls into PA intervention RCTs. Teacher involvement consistently boosted recruitment – especially when teachers delivered programmes or were financially supported – while incentives for participants undermined both recruitment and retention. Provided ethical standards are upheld, these findings may guide PA programme providers, researchers, and funders in smarter resource allocation.

Demographics mattered: recruitment was lower among girls from low-SES backgrounds and older adolescents, pointing to the need for targeted strategies in these groups. Recruitment improved when programmes were embedded into curriculum time and when researchers actively engaged girls through presentations or taster sessions. A wide range of PA types were acceptable, suggesting tailoring to local microcultures may be key.

Overall, effective recruitment may hinge on leveraging teacher involvement, integrating PA within school schedules, aligning activities with girls’ preferences, and raising reporting standards to strengthen the evidence base.

### Future research

Future work should prioritise standardised reporting of recruitment metrics and inclusion of diverse socioeconomic and geographic contexts to strengthen generalisability. Furthermore, future work might benefit from experimental or quasi-experimental comparisons of recruitment strategies, in which the strategies themselves are randomised. Future work might also explore how PA type influences both recruitment and retention, and clarify how BMI-focused aims impact participation. Sensitivity findings suggest that feasibility and fully powered studies may yield different recruitment patterns; future reviews should stratify analyses accordingly. Beyond RCTs, investigating recruitment into real-world PA programmes would further inform effective strategies to engage adolescent girls.

## Supplementary Information

Supplementary Material 1.

## Data Availability

The datasets used and/or analysed during the current study are available from the corresponding author on reasonable request.

## Abbreviations

BMI: Body mass index
PA: Physical activity
PICOTS: Population, Intervention, Comparison, Outcome, Time frame, Study Type
PRISMA: Preferred Reporting Items for Systematic Reviews and Meta-Analysis
RCT: Randomised controlled trial
SEAR: Screened, Eligible, Approached, Randomised
SES: Socioeconomic status
VIF: Variance inflation factors
WHO: World Health Organisation
