# Supplementary material for: Strategies for recruitment of adolescent girls into physical activity programmes: a systematic review and regression analysis
Source: BMC Public Health. 2026 Apr 11;26:1647. doi: 10.1186/s12889-026-27261-z (PMC13196039; doi:10.1186/s12889-026-27261-z)
Supplement: Supplementary file 1 — Supplementary Material 1. [file 12889_2026_27261_MOESM1_ESM.docx]

**Additional Files**

**Item 1: Prisma 2020 Checklist [25]**

| **Section and Topic** | **Item #** | **Checklist item** | **Location where item is reported** |
| --- | --- | --- | --- |
| **TITLE** | | |  |
| Title | 1 | Identify the report as a systematic review. | Title |
| **ABSTRACT** | | |  |
| Abstract | 2 | See the PRISMA 2020 for Abstracts checklist. | Supplementary materials |
| **INTRODUCTION** | | |  |
| Rationale | 3 | Describe the rationale for the review in the context of existing knowledge. | Introduction |
| Objectives | 4 | Provide an explicit statement of the objective(s) or question(s) the review addresses. | Objectives |
| **METHODS** | | |  |
| Eligibility criteria | 5 | Specify the inclusion and exclusion criteria for the review and how studies were grouped for the syntheses. | Eligibility criteria |
| Information sources | 6 | Specify all databases, registers, websites, organisations, reference lists and other sources searched or consulted to identify studies. Specify the date when each source was last searched or consulted. | Timeframe, Information sources and search strategy |
| Search strategy | 7 | Present the full search strategies for all databases, registers and websites, including any filters and limits used. | Supplementary material |
| Selection process | 8 | Specify the methods used to decide whether a study met the inclusion criteria of the review, including how many reviewers screened each record and each report retrieved, whether they worked independently, and if applicable, details of automation tools used in the process. | Data management, Search and selection |
| Data collection process | 9 | Specify the methods used to collect data from reports, including how many reviewers collected data from each report, whether they worked independently, any processes for obtaining or confirming data from study investigators, and if applicable, details of automation tools used in the process. | Data extraction |
| Data items | 10a | List and define all outcomes for which data were sought. Specify whether all results that were compatible with each outcome domain in each study were sought (e.g. for all measures, time points, analyses), and if not, the methods used to decide which results to collect. | Data extraction |
|  | 10b | List and define all other variables for which data were sought (e.g. participant and intervention characteristics, funding sources). Describe any assumptions made about any missing or unclear information. | Data extraction |
| Study risk of bias assessment | 11 | Specify the methods used to assess risk of bias in the included studies, including details of the tool(s) used, how many reviewers assessed each study and whether they worked independently, and if applicable, details of automation tools used in the process. | N/A: Design |
| Effect measures | 12 | Specify for each outcome the effect measure(s) (e.g. risk ratio, mean difference) used in the synthesis or presentation of results. | Data synthesis |
| Synthesis methods | 13a | Describe the processes used to decide which studies were eligible for each synthesis (e.g. tabulating the study intervention characteristics and comparing against the planned groups for each synthesis (item #5)). | Data synthesis |
|  | 13b | Describe any methods required to prepare the data for presentation or synthesis, such as handling of missing summary statistics, or data conversions. | Data synthesis |
|  | 13c | Describe any methods used to tabulate or visually display results of individual studies and syntheses. | Data synthesis |
|  | 13d | Describe any methods used to synthesize results and provide a rationale for the choice(s). If meta-analysis was performed, describe the model(s), method(s) to identify the presence and extent of statistical heterogeneity, and software package(s) used. | Design, Data synthesis |
|  | 13e | Describe any methods used to explore possible causes of heterogeneity among study results (e.g. subgroup analysis, meta-regression). | Design, Data synthesis |
|  | 13f | Describe any sensitivity analyses conducted to assess robustness of the synthesized results. | Data synthesis - Sensitivity analysis |
| Reporting bias assessment | 14 | Describe any methods used to assess risk of bias due to missing results in a synthesis (arising from reporting biases). | N/A |
| Certainty assessment | 15 | Describe any methods used to assess certainty (or confidence) in the body of evidence for an outcome. | N/A |
| **RESULTS** | | |  |
| Study selection | 16a | Describe the results of the search and selection process, from the number of records identified in the search to the number of studies included in the review, ideally using a flow diagram. | Search and selection |
|  | 16b | Cite studies that might appear to meet the inclusion criteria, but which were excluded, and explain why they were excluded. | Limitations |
| Study characteristics | 17 | Cite each included study and present its characteristics. | Characteristics of included studies |
| Risk of bias in studies | 18 | Present assessments of risk of bias for each included study. | N/A: Design |
| Results of individual studies | 19 | For all outcomes, present, for each study: (a) summary statistics for each group (where appropriate) and (b) an effect estimate and its precision (e.g. confidence/credible interval), ideally using structured tables or plots. | Recruitment and retention rates, Effectiveness of recruitment strategies and programme design factors |
| Results of syntheses | 20a | For each synthesis, briefly summarise the characteristics and risk of bias among contributing studies. | Recruitment and retention rates, Effectiveness of recruitment strategies and programme design factors |
|  | 20b | Present results of all statistical syntheses conducted. If meta-analysis was done, present for each the summary estimate and its precision (e.g. confidence/credible interval) and measures of statistical heterogeneity. If comparing groups, describe the direction of the effect. | Recruitment and retention rates, Effectiveness of recruitment strategies and programme design factors |
|  | 20c | Present results of all investigations of possible causes of heterogeneity among study results. | Recruitment and retention rates, Effectiveness of recruitment strategies and programme design factors |
|  | 20d | Present results of all sensitivity analyses conducted to assess the robustness of the synthesized results. | Results – Sensitivity analysis |
| Reporting biases | 21 | Present assessments of risk of bias due to missing results (arising from reporting biases) for each synthesis assessed. | N/A |
| Certainty of evidence | 22 | Present assessments of certainty (or confidence) in the body of evidence for each outcome assessed. | N/A |
| **DISCUSSION** | | |  |
| Discussion | 23a | Provide a general interpretation of the results in the context of other evidence. | Recruitment rates for adolescent girls into PA RCTs, Effectiveness of recruitment strategies to enrol adolescent girls in PA RCTs |
|  | 23b | Discuss any limitations of the evidence included in the review. | Limitations |
|  | 23c | Discuss any limitations of the review processes used. | Limitations |
|  | 23d | Discuss implications of the results for practice, policy, and future research. | Effectiveness of recruitment strategies to enrol adolescent girls in PA RCTs, Conclusion |
| **OTHER INFORMATION** | | |  |
| Registration and protocol | 24a | Provide registration information for the review, including register name and registration number, or state that the review was not registered. | Methods |
|  | 24b | Indicate where the review protocol can be accessed, or state that a protocol was not prepared. | Methods |
|  | 24c | Describe and explain any amendments to information provided at registration or in the protocol. | Results |
| Support | 25 | Describe sources of financial or non-financial support for the review, and the role of the funders or sponsors in the review. | End of manuscript |
| Competing interests | 26 | Declare any competing interests of review authors. | End of manuscript |
| Availability of data, code and other materials | 27 | Report which of the following are publicly available and where they can be found: template data collection forms; data extracted from included studies; data used for all analyses; analytic code; any other materials used in the review. | End of manuscript |

*From:*  Page MJ, McKenzie JE, Bossuyt PM, Boutron I, Hoffmann TC, Mulrow CD, et al. The PRISMA 2020 statement: an updated guideline for reporting systematic reviews. BMJ 2021;372:n71. doi: 10.1136/bmj.n71. This work is licensed under CC BY 4.0. To view a copy of this license, visit <https://creativecommons.org/licenses/by/4.0/>

**Item 2: Search Strategy**

EMBASE (2354)

'girl'/exp OR ('female'/exp AND ('adolescent'/de OR 'adolescent health'/exp))

Girl*:ti,ab,kw

(female* NEAR/3 (teen* OR adolescen* OR child* OR school-age)):ti,ab,kw

#1 OR #2 OR #3

((recruit* OR enrol* OR promot* OR participat* OR engag*) NEAR/5 (sport* OR 'physical activity' OR exercis* OR fitness)):ti,ab,kw

((recruit* OR enrol* OR promot* OR participat* OR engag*) NEAR/5 (girl* OR female OR gender*)):ti,ab,kw

((increas* OR 'take part' OR involv* OR improve) NEAR/3 (sport* OR 'physical activity' OR exercis*)):ti,ab,kw

#5 OR #6 OR #7

'exercise'/exp OR 'kinesiotherapy'/exp OR 'physical activity'/exp OR 'physical activity, capacity and performance'/de OR 'training'/de OR 'endurance'/de OR 'exercise tolerance'/de OR 'physical capacity'/de OR 'sport'/exp

(exercis* OR isometric* or isotonic* or isokinetic*):ti,ab,kw

(resistance OR strength* OR weight* OR endurance OR sport*):ti,ab,kw

(physical* NEAR/3 (fit* or therap* OR activity)):ti,ab,kw

#9 OR #10 OR #11 OR #12

'clinical trial'/de OR 'randomized controlled trial'/de OR 'randomization'/de OR 'single blind procedure'/de OR 'double blind procedure'/de OR 'crossover procedure'/de OR 'placebo'/de OR 'prospective study'/de OR ('randomi?ed controlled' NEXT/1 trial*) OR rct OR 'randomly allocated' OR 'allocated randomly' OR 'random allocation' OR (allocated NEAR/2 random) OR (single NEXT/1 blind*) OR (double NEXT/1 blind*) OR ((treble OR triple) NEAR/1 blind*) OR placebo*

#4 AND #8 AND #13 AND #14

Medline [2272]

Randomized controlled trials as Topic/

Randomized controlled trial/

Random allocation/

Double blind method/

Single blind method/

Clinical trial/

exp Clinical Trials as Topic/

or/1-7

(clinic$ adj trial$1).tw.

((singl$ or doubl$ or treb$ or tripl$) adj (blind$3 or mask$3)).tw.

Placebos/

Placebo$.tw.

Randomly allocated.tw.

(allocated adj2 random).tw.

or/9-14

8 or 15

Case report.tw.

Letter/

Historical article/

Review of reported cases.pt.

Review, multicase.pt.

or/17-21

16 not 22

(single arm OR phase 2 OR phase II OR prospective).ti,ab.

23 OR 24

(exp Women/ OR Female/) AND (Adolescent/ OR Adolescent Behavior/ OR exp Child/)

Girl*.ti,ab.

(female* adj3 (teen* OR adolescen* OR child* OR school-age)).ti,ab.

or/24-26

((recruit* OR enrol* OR promot* OR participat* OR engag*) adj5 (sport* OR physical activity OR exercis* OR fitness)).ti,ab.

((recruit* OR enrol* OR promot* OR participat* OR engag*) adj5 (girl* OR female OR gender*)).ti,ab.

((increas* OR take part OR involv* OR improve) adj3 (sport* OR physical activity OR exercis*)).ti,ab.

or/28-30

exp Exercise/ OR exp Exercise Therapy/ OR exp Physical Fitness/ OR exp "physical education and training"/ OR exp "Exercise Movement Techniques"/ or physical endurance/ or exercise tolerance/ OR Physical Exertion/ or exp Sports/ or Dancing/

(strength* or isometric* or isotonic* or isokinetic* or exercis*).ti,ab.

(resistance adj3 train*).ti,ab.

((physical* or motion* or cardiopulmonary or cardiorespiratory) adj3 (fit* or therap* or activit*)).ti,ab.

(treadmill* or cross-train* or rowing or sport* or exercise* or physical* activit* or aerobic* or run or jog* or running or walk or walks or walking or gym* or yoga or pilates or "recreation* activit*" or zumba or salsa* or cycling or bicycle or bike or swim* or dance or dancer* or dances or dancing or physiotherapy* or physical therap*).ti,ab.

(circuit* adj1 train*).ti,ab.

(keep* adj1 (active or fit)).ti,ab.

or/32-38

23 and 27 and 31 and 39

CINAHL [2785]

(MH "Female") AND (MH "Adolescence" OR MH "Child" OR MH "Adolescent Psychology" OR MH "Adolescent Behavior")

TI (Girl*) OR AB (Girl*)

TI (female* N3 (teen* OR adolescen* OR child* OR school-age)) OR AB (female* N3 (teen* OR adolescen* OR child* OR school-age))

S1 OR S2 OR S3

TI ((recruit* OR enrol* OR promot* OR participat* OR engag*) N5 (sport* OR "physical activity" OR exercis* OR fitness)) OR AB ((recruit* OR enrol* OR promot* OR participat* OR engag*) N5 (sport* OR "physical activity" OR exercis* OR fitness))

TI ((recruit* OR enrol* OR promot* OR participat* OR engag*) N5 (girl* OR female OR gender*)) OR AB ((recruit* OR enrol* OR promot* OR participat* OR engag*) N5 (girl* OR female OR gender*))

TI ((increas* OR 'take part' OR involv* OR improve) N3 (sport* OR "physical activity" OR exercis*)) OR AB ((increas* OR 'take part' OR involv* OR improve) N3 (sport* OR "physical activity" OR exercis*))

S5 OR S6 OR S7

(MH "Exercise+") OR (MH "Physical Activity") OR (MH "Physical Fitness+") OR (MH "Physical Performance") OR (MH "Sports+") OR (MH "Resistance Training") OR (MH "Therapeutic Exercise+") OR (MH "Exercise Intensity")

TI (isometric* or isotonic* or isokinetic* OR exercis*) OR AB (isometric* or isotonic* or isokinetic* OR exercis*)

TI ((resistance OR strength* OR weight* OR endurance) N3 (train* OR exercis*)) OR AB ((resistance OR strength* OR weight* OR endurance) N3 (train* OR exercis*))

TI ((physical* or motion* or cardiopulmonary or cardiorespiratory) N3 (fit* or therap*)) OR AB ((physical* or motion* or cardiopulmonary or cardiorespiratory) N3 (fit* or therap*))

TI (treadmill* or cross-train* or rowing or sport* OR exercise* OR "physical activit*" OR aerobic* OR run or jog* or running OR walk or walks or walking OR gym* OR pilates OR "recreation* activit*" OR cycling or bicycle or bike or swim*) OR AB (treadmill* or cross-train* or rowing or sport* OR exercise* OR "physical activit*" OR aerobic* OR run or jog* or running OR walk or walks or walking OR gym* OR pilates OR "recreation* activit*" OR cycling or bicycle or bike or swim*)

TI (circuit* N1 train*) OR AB (circuit* N1 train*)

TI (keep* N1 (active or fit)) OR AB (keep* N1 (active or fit))

S9 OR S10 OR S11 OR S12 OR S13 OR S14 OR S15

TX allocat* random* OR (MH "Quantitative Studies") OR (MH "Placebos") OR TX placebo* OR TX random* allocat* OR (MH "Random Assignment") OR TX randomi* control* trial* OR TX ( (singl* n1 blind*) OR (singl* n1 mask*) ) OR TX ( (doubl* n1 blind*) OR (doubl* n1 mask*) ) OR TX ( (tripl* n1 blind*) OR (tripl* n1 mask*) ) OR TX ( (trebl* n1 blind*) OR (trebl* n1 mask*) ) OR TX clinic* n1 trial* OR PT Clinical trial OR (MH "Clinical Trials+")

S4 AND S8 AND S16 AND S17

Web of Science [590]

Girl* OR (female* NEAR/3 (teen* OR adolescen* OR child* OR school-age))

((recruit* OR enrol* OR promot* OR participat* OR engag*) NEAR/5 (sport* OR "physical activity" OR exercis* OR fitness)) OR ((recruit* OR enrol* OR promot* OR participat* OR engag*) NEAR/5 (girl* OR female OR gender*)) OR ((increas* OR "take part" OR involv* OR improve) NEAR/3 (sport* OR "physical activity" OR exercis*))

((strength* or isometric* or isotonic* or isokinetic* or exercis* OR treadmill* or cross-train* or rowing or sport* or exercise* or "physical* activit*" or aerobic* or run or jog* or running or walk or walks or walking or gym* or yoga or pilates or "recreation* activit*" or zumba or salsa* or cycling or bicycle or bike or swim* or dance or dancer* or dances or dancing or physiotherapy*) OR (resistance NEAR/3 train*) OR ((physical* or motion* or cardiopulmonary or cardiorespiratory) NEAR/3 (fit* or therap* or activit*)) OR (circuit* NEAR/1 train*) OR (keep* NEAR/1 (active or fit)))

(randomised OR randomized OR randomisation OR randomisation OR placebo* OR (random* AND (allocat* OR assign*)) OR (blind* AND (single OR double OR treble OR triple)))

#1 AND #2 AND #3 AND #4

Cochrane Library – Central Trial Registry [2049]

([mh Women] OR [mh Female]) AND ([mh Adolescent] OR [mh "Adolescent Behavior"] OR [mh Child])

Girl*:ti,ab,kw

(female* NEAR/3 (teen* OR adolescen* OR child* OR school-age)):ti,ab,kw

#1 OR #2 OR #3

((recruit* OR enrol* OR promot* OR participat* OR engag*) NEAR/5 (sport* OR "physical activity" OR exercis* OR fitness)):ti,ab,kw

((recruit* OR enrol* OR promot* OR participat* OR engag*) NEAR/5 (girl* OR female OR gender*)):ti,ab,kw

((increas* OR take part OR involv* OR improve) NEAR/3 (sport* OR "physical activity" OR exercis*)):ti,ab,kw

#5 OR #6 OR #7

[mh "exercise"] OR [mh "kinesiotherapy"] OR [mh "physical activity"] OR [mh ^"physical activity, capacity and performance"] OR [mh ^Training] OR [mh ^Endurance] OR [mh ^"exercise tolerance"] OR [mh ^"physical capacity"] OR [mh "sport"]

(exercis* OR isometric* or isotonic* or isokinetic*):ti,ab,kw

((resistance OR strength* OR weight* OR endurance) NEAR/3 (train* OR exercise*)):ti,ab,kw

((physical* or motion* or cardiopulmonary or cardiorespiratory) NEAR/3 (fit* or therap*)):ti,ab,kw

(treadmill* or cross-train* or rowing or sport* OR exercise* OR "physical activit*" OR aerobic* OR run or jog* or running OR walk or walks or walking OR gym* OR yoga oR pilates OR "recreation* activit*" OR cycling or bicycle or bike or swim* OR HIIT OR weightlift* OR weight-lift*):ti,ab,kw

(circuit* NEAR/1 train*):ti,ab,kw

(keep* NEAR/1 (active or fit)):ti,ab,kw

#9 OR #10 OR #11 OR #12 OR #13 OR #14 OR #15

#4 AND #8 AND #16

**Item 3: List of Variables**

The following data was extracted:

| **Category** | **Variables** | **Variable Type** | **Predictor Type** |
| --- | --- | --- | --- |
| **Demographics (Independent variables)** | PA programme name  Year of publication  Country  Participants’ mean age  Socioeconomic status (high/mixed/low)  Urban or rural setting | Nominal  Ordinal  Nominal  Continuous  Ordinal  Binary | Independent |
| **Study design** | Individual or cluster RCT  Feasibility study or full RCT  One PA type or a variety  PA-only programme or multicomponent  Based on underpinning theory (yes/no)* | Binary  Binary  Binary  Binary  Binary | Independent |
| **Dosage of PA programme** | Length of programme in weeks  Duration of each session in minutes  Number of sessions per week  Trimmed number of sessions per week  Number of participants randomised  Trimmed number of participants randomised | Continuous  Continuous  Continuous  Continuous  Continuous  Continuous | Independent  Independent  Independent  Independent  **Dependent**  **Dependent** |
| **Recruitment Statistics** | Recruitment rate %*  Met or did not meet 100% of pre-determined recruitment goal*  % of recruitment goal achieved*  Recruitment challenges were identified by the authors (yes/no) | Continuous  Continuous  Continuous  Binary | **Dependent** |
| **Retention Statistics** | Retention Rate %*  Attendance challenges were identified by the authors (yes/no) | Continuous  Binary | **Dependent** |
| **Programme location & timing** | Programme offered before school/during lunch/after school*  Programme replaced traditional PE class on school property  Programme offered through local community PA locations  Programme offered online or remotely*  Located on school property or not on school property | Binary  Binary  Binary  Binary  Binary | Independent |
| **Programme type** | Walking  Yoga/dance/pilates/Zumba/aerobics*  General fitness/resistance training/FMS/HIIT*  Individual sports*  Team sports* | Binary  Binary  Binary  Binary  Binary | Independent |
| **Instructor** | School-based instructor*  Non-school-based instructor* | Binary  Binary | Independent |
| **Recruitment strategies** | Incentives*  Active school/teacher recruiting*  Active researcher recruiting*  Passive school recruiting*  Parent/Community/Online recruiting* | Binary  Binary  Binary  Binary  Binary | Independent |
| **Measurement** | Accelerometers or pedometers worn  Fitness testing*  Body measurements*  Medical testing* | Binary  Binary  Binary  Binary | Independent |
| **Effectiveness** | Was the programme effective at increasing PA levels? (yes/no) | Binary | Independent |

*indicates that this variable was condensed for clarity and practicality of analysis. See the below table for further details.

The following original variables were condensed for clarity and practicality of analysis:

| **Condensed Variable Description** | **Original Variable Description** |
| --- | --- |
| **Recruitment Rate % (Approached or Screened/Randomised)** | Number of Potential Participants Screened |
|  | Number of Participants Eligible |
|  | Number of Participants Approached |
|  | Number of Participants Randomised |
| **Met or did not meet 100% of Pre-Determined Recruitment Goal** | Pre-Determined Recruitment Goal |
|  | Number of Participants Randomised |
|  | Met Pre-Determined Recruitment Goal |
| **%of Recruitment Goal Achieved (Number Randomised/Recruitment Goal X 100%)** | Number of Participants Randomised |
|  | Pre-Determined Recruitment Goal |
| **Retention Rate % of Participants (Retained/Randomised)** | Number of Participants Retained to End of Study |
|  | Number of Participants Randomised |
| **Programme Offered Before, at Lunchtime, or After School, on school property** | Programme Offered Before School, on School Property |
|  | Programme Offered Before During Lunchtime, on School Property |
|  | Programme Offered After School, on School Property |
| **Programme Offered Online or Remotely** | Programme Offered Online |
|  | Programme able to be Completed by Participant at Anytime, in Any Location |
| **Yoga, Dance, Pilates, Zumba, Aerobics** | Yoga |
|  | Pilates |
|  | Zumba |
|  | Dance |
|  | Aerobics |
| **General Fitness, Resistance Training, FMS, HIIT** | Resistance Training |
|  | General Fitness Activities, Cardiovascular and Resistance Training |
|  | Functional Movement Scale Training |
|  | High Intensity Interval Training |
| **Individual Activities** | Skipping |
|  | Boxing |
|  | Canoeing |
|  | Martial Arts and/or Self-Defense |
|  | Kickboxing |
|  | Running |
| **Team Activities** | Soccer |
|  | Games |
|  | Basketball |
|  | Touch Football |
|  | Volleyball |
|  | Handball |
|  | Tennis |
|  | Aquatic Games |
| **Based on Underpinning Theory** | Self-Determination Theory |
|  | Social Cognitive Theory |
|  | Behaviour Change Technique Taxonomy |
|  | Socio-Ecological Model |
|  | Operant Learning Theory |
|  | Organizational Change Theory |
|  | Health Promotion Model |
|  | Coordinated School Health Program Model |
|  | Health Promoting Schools Framework |
|  | Action Learning Approach |
|  | Acceptance and Commitment Therapy Model |
|  | Game Sense |
|  | Capacity Building Framework |
|  | Expectancy-Value Theory |
|  | Promoting Exercise for Physical and Mental Health Conceptual Framework |
|  | Competence Motivation Theory |
|  | Trans-Theoretical Model |
|  | Self-Determination Theory |
|  | Social Cognitive Theory |
|  | Behaviour Change Technique Taxonomy |
|  | Socio-Ecological Model |
|  | Operant Learning Theory |
|  | Organizational Change Theory |
|  | Health Promotion Model |
|  | Coordinated School Health Program Model |
|  | Health Promoting Schools Framework |
|  | Action Learning Approach |
|  | Acceptance and Commitment Therapy Model |
|  | Game Sense |
|  | Capacity Building Framework |
|  | Expectancy-Value Theory |
|  | Promoting Exercise for Physical and Mental Health Conceptual Framework |
|  | Competence Motivation Theory |
|  | Trans-Theoretical Model |
| **School-based instructor** | PA Programme Delivered by Peers |
|  | PA Programme Delivered by Teachers |
|  | PA Programme Delivered by Research who is a Teacher at the School |
| **Non-school-based instructor** | PA Programme Delivered by Near-Peers (Usually undergraduate students) |
|  | PA Programme Delivered by Community Fitness Instructors |
|  | PA Programme Delivered by Member of Research Team |
|  | PA Programme Delivered by Physical Education Graduates |
| **Fitness testing** | Fitness Testing |
|  | Functional Movement Testing |
| **Body measurements** | Objectively measured Height and Weight |
|  | Objectively measured Waist and Hip Circumference |
|  | Objectively measured Body Fat Percentage |
| **Medical testing** | Sexually Transmitted Infection Testing |
|  | Pregnancy Testing |
|  | Bloodwork drawn for Testing |
|  | Blood Pressure Testing |
|  | DXA scan for Bone Mineral Density |
|  | Urine Test |
| **Incentives** | Study Branded Clothing provided to Participants |
|  | Participants are Paid for Participation |
|  | Participants have the Opportunity to win Prizes for Participation in a Draw |
|  | Participants are given small gifts for participation |
|  | Participants may earn small prizes for participation |
|  | Participants have opportunities to earn Awards or Certificates through Participation |
|  | Gift or Food Vouchers provided for Participation |
|  | Potential Participants were paid for return of Consent Forms whether they were interested or not |
| **Active school/teacher recruiting** | Schools and/or teachers are provided with payment for assisting with the study |
|  | Teachers Recruited Potential Participants Directly |
|  | Follow-up with Absent Students or Non-Returned forms in Schools |
| **Active researcher recruiting** | Researchers Present Directly to Potential Participants in School |
|  | A Taster Session was provided to all Potentially Eligible Students in Schools |
| **Passive school recruiting** | Flyers Posted in Schools |
|  | Information Packages and Consent Forms Distributed to all Potential Participants in Schools |
|  | Advertised in School Newsletter |
|  | Recruitment Video |
| **Parent/Community/Online recruiting** | Researcher presentation to Parents |
|  | Mass Mailing of Study Information Directly to Parents |
|  | Participants Recruited through their Church |
|  | Paid Advertising of Study on Instagram |

*Self-report questionnaires were not included as a measurement variable because every study included self-report questionnaires

**Item 4: Significant Correlation results**

Significant correlation results

| **Variable 1** | **Variable 2** | **Pearson correlation** | **Sig (2-tailed)** | **n** |
| --- | --- | --- | --- | --- |
| **Recruitment rate** | **All studies** | | | |
|  | Programme offered online or remotely (use of activity trackers) | .509 | .031* | 18 |
|  | Individual activities (i.e. running, skipping, boxing, canoeing, martial arts) | .676 | .002** | 18 |
|  | Programme offered remotely (activity trackers) | .509 | .031* | 18 |
|  | Aim to improve BMI | .555 | .017* | 18 |
|  | **School-based studies** | | | |
|  | Individual activities | .790 | <.001** | 15 |
|  | Aim to improve BMI | .668 | .006** | 15 |
|  | Teacher instructor | .532 | .041* | 15 |
|  | **All studies** | | | |
| **% of recruitment goal** | Programme offered on school property | .504 | .033* | 18 |
|  | Programme offered online/anytime | -.528 | .024* | 18 |
|  | Programme offered remotely using activity trackers | -.528 | .024* | 18 |
|  | Soccer | .471 | .042* | 19 |
|  | Basketball | .506 | .027* | 19 |
|  | Tennis | .528 | .020 | 19 |
|  | Aim to reduce depression | -.682 | .001** | 19 |
|  | Schools/teachers paid | .568 | .022* | 16 |
|  | Presentation to parents | -.695 | .003** | 16 |
|  | Recruited through church | -.695 | .003** | 16 |
|  | **School-based studies** | | | |
|  | Basketball | .552 | .033* | 15 |
|  | Tennis | .597 | .019* | 15 |
|  | Aim to decrease sedentary time | -.515 | .049* | 15 |
|  | Schools/teachers paid | .677 | .011* | 13 |
| **Retention rate** | **All studies** | | | |
|  | Yoga/dance/pilates/Zumba/aerobics | -.481 | .023* | 22 |
|  | Fitness or FMS assessments | .493 | .020* | 22 |
|  | Programme offered online (*only one programme) | -.489 | .024* | 21 |
|  | Dance | -.635 | .002** | 22 |
|  | Delivered by Community Fitness Instructors | -.470 | .031* | 21 |
|  | Paid advertising on Instagram | -.532 | .034* | 16 |
|  | **School-based studies** | | | |
|  | Yoga/Dance/Pilates/Zumba/Aerobics | -.496 | .043* | 17 |
|  | Dance | -.605 | .010* | 17 |
|  | Community Fitness Instructors | -.496 | .043* | 17 |
|  | Recruitment Video | -.640 | .019* | 13 |
|  | Study Clothing | -.640 | .019* | 13 |
|  | Gifts for participation | -.620 | .024* | 13 |
|  | Paid for Consent form return | -.640 | .019* | 13 |
